# Nursing Progress in America

**Published:** 1917-04-28

**Authors:** 


### THE MATRONS' AND SISTERS' DEPARTMENT.

### NURSING PROGRESS IN AMERICA.

By A GRADUATE OF TEACHER'S COLLEGE.

It cannot fail to be of interest to see how nurses are working towards true State service in the United States of America, now allied to Great Britain, which for many years has taken democracy as its keynote. Here the aspirations, ideals, and finest traditions of nursing are focussed at the Department of Nursing and Health, Teacher's College, Columbia University, New York.

In speaking of this College Course for Nurses, a Professor of Columbia University has said: "The University should be peculiarly interested in providing for the highest and most selective training of those who are to engage in the pursuits by which human life, human development, and human health are concerned. The future teacher—sanitarian—physician and nurse should be among the specially chosen subjects of its educational care and culture, by virtue of the very nature and purpose of the offices they are elected to fill. To win for herself so fitting a place as the handmaid of modern and preventive medicine, to hold for herself her traditional place in the ministry of human pain, the nurse of to-day can neither be too wise nor too womanly, too trained or too good."

Apart from the spirit of progress and breadth of vision to be gleaned from a course at Teacher's College, the dominant note of the Nursing and Health Department, as in so many other departments of American affairs, is essentially organisation, and it is within the College itself, taking lectures with students in the fine arts, household arts and educational supervision, that is felt the value and importance of nursing in relation to public welfare and the health of the community, also the importance of the reaction of the education a nurse receives, not only upon the hospital and the medical world, but upon the general public, whether sick or in health; and it is from this socialising aspect that most of the students taking this course derive most benefit.

The Department of Nursing and Health at Teacher's College may be divided into:—

(A) The Department for the Teaching and Supervision in Training Schools for Nurses. Here it is interesting to note the number of positions held involving little or no executive work, but demanding trained teachers for instruction alone.

(B) The Lectures by responsible Superintendents of Nurses on Administration in Hospitals and Training-Schools where difficulties and problems are discussed. The opportunities afforded the students to visit various hospitals and institutions, with full facilities to see how administrators are meeting, and overcoming, their difficulties.

(C) The Social and Preventive Branches of Nursing, including Visiting Nursing, School and Municipal Nursing, and Sanitary Inspection. For this field of work, courses in the College are supplemented by work in the School of Philanthropy, Social Settlements, and the Municipal Health Department.

(D) Special work offered to students at Teacher's College in the preliminary sciences leading to nursing, before they are of an age to be trained as nurses. Some of the subjects studied being anatomy, physiology, bacteriology, chemistry, household economics, cookery, laundry, home nursing, and lectures upon the history of nursing and hospital and nursing ethics. In the United States it is held that such a foundation could not be other than advantageous to the patient, the staff, and the individual probationers, preventing nervous strain and giving the latter a better understanding of their immediate duties. This preliminary course takes eight months, and many of the leading hospitals in America have agreed to deduct six to nine months from the regular course of the student-nurse who has received this preparation.

It will be seen, therefore, that in the United States, under A, B, C, and D, provision has been made for the trained nurse who wishes to specialise in certain branches of the profession, and for the untrained woman previous to her entering it. Psychology is a compulsory subject for every student entering the College.

The fact that at least fifteen of the training-schools in America are in direct relationship to Universities speaks well for the progressive spirit in the profession there. The most prominent effort is to be seen in the School of Nursing at the University of Minnesota, where the preliminary course of instruction and its undergraduate lectures are given in the laboratories and lecture-rooms of the College, its examinations conducted by the chief of the College Departments, and its diplomas conferred by the regents of the University. The University of Minnesota is devoted to the education of medical students and the training of nurses, and thus realises one of the highest ideals of a hospital service.

A similar arrangement has also been made at the University of Texas, where the pupil-nurses are recognised as students of the Medical Department of the University, the clinical instructor of nursing being the superintendent of the nurses. The obvious advantage of paid instructors, libraries, laboratories, and teaching material, with necessitated hours of study, all tend to attract a finer and more efficient type of woman into the profession. Here, therefore, we see women working shoulder to shoulder with men, and America's wisdom in recognising so early the contribution women have to make to State service.

The Royal College of British Nursing now in process of organisation should speedily secure similar advantages and recognition for trained nurses in Great Britain. British nurses have been stimulated by the country's present great need, which tradition has too long left unsupplied. This delay will afford the Royal College of British Nursing a magnificent opportunity to develop many latent possibilities for the advancement of the Profession of British Nursing, and fully trained nurses, throughout the United Kingdom.

